# Research on Pedestrian Detection Algorithm Based on MobileNet-YoLo

**DOI:** 10.1155/2022/8924027

**Published:** 2022-10-30

**Authors:** Lisang Liu, Chengyang Ke, He Lin, Hui Xu

**Affiliations:** ^1^School of Electronic Electrical Engineering and Physics, Fujian University of Technology, Fuzhou, Fujian, China; ^2^State Grid Fujian Power Supply Co. LTD., Xiapu Power Supply Company, Ningde, Fujian, China

## Abstract

To address the problem that large pedestrian detection networks cannot be directly applied to small device scenarios due to the heavyweight and slow detection speed, this paper proposes a pedestrian detection and recognition model MobileNet-YoLo based on the YoLov4-tiny target detection framework. To address the problem of low accuracy of YoLov4-tiny, MobileNetv3 is used to optimize its backbone feature extraction network, and the MFF model is proposed to fuse the output of the first two layers to solve the information loss problem, and the attention mechanism CBAM is introduced after strengthening the feature extraction network to further improve the detection efficiency; then the 3 × 3 convolution is replaced by the depth separable convolution, which greatly reduces the number of parameters and thus improves the detection rate, then propose Ordinary data augmentation to efficiently augment the dataset and dynamically adjust the target detection anchor frame using the k-means++ clustering algorithm. Finally, the model weights trained by the VOC2007 + 2012 dataset were applied to the pedestrian dataset for retraining by the transfer learning method, which effectively solved the problem of scarce samples and greatly shortened the training time. The experimental results on the VOC2007 + 2012 dataset show that the average means accuracy of the MobileNet-YoLo model compared to YoLov4-tiny, MobileNet-YoLov4, MobileNet-YoLov3, and YoLov5s by 5.00%, 1.30%, 3.23%, and 0.74%, respectively and have reached the level to realize the landed application.

## 1. Introduction

Pedestrian detection [1–3] has become one of the most important research directions in the field of computer vision and an important research topic in deep learning, moreover, the core technology relied on security, intelligent video surveillance, and scenic traffic statistics, and its accuracy is of great significance to the development of security intelligent video surveillance systems. Surveillance cameras are widely installed in public places, and pedestrian detection can be achieved with the help of big data [4–7] and other IoT systems [8–11]. Although current detection algorithms have developed better in terms of detection accuracy and speed, pedestrian detection still faces many challenges. For example, when multiple pedestrians are present in the same scene, two or more pedestrians can block each other, and accurate detection is still difficult. These uncertainties make the research of pedestrian detection techniques exceptionally difficult.

Before the advent of machine learning [12–14] techniques, the task of pedestrian detection in surveillance videos was mostly carried out manually, which required a lot of human and material resources, and much of the information in the videos could not be well understood. With continuous breakthroughs in computer vision and machine learning technologies, pedestrian detection methods based on shallow learning have emerged. Traditional target detection algorithms use a sliding window strategy to select candidate regions on a given image, extract features from these regions [15, 16], and finally use a trained classifier [17] for classification. This method has disadvantages such as low relevance, high time complexity, and poor robustness. Gong [18] used a pedestrian detection model based on hybrid Gaussian and HOG + SVM to identify the type of information contained in foreground images and reduce the false detection rate, but the background modeling is more complex; Chen [19] used HOG features and machine learning for pedestrian detection and used edge detection to quickly detect the candidate areas of travelers and improve the detection rate, but the real-time performance is poor. However, building a deep neural network [20] can effectively grasp the deep features of images through self-learning to further solve the above problems.

At present, deep learning-based target detection algorithms are becoming more and more mature and can be divided into two categories: one-stage detection algorithms and two-stage detection algorithms. One-stage algorithms mainly include YoLo series algorithms [21–23], SSD algorithms [24–26], and RetinaNet algorithms [27]. Due to the slow detection speed and relatively large weight of YoLov4-tiny, Zhou [28] proposed three models for large pedestrian detection and recognition, and all three models improved in actual detection accuracy compared with the original model, which not only satisfied the lightweight but also its detection speed did not decrease very significantly. In the literature [29], YoLov3 was simplified and the feature fusion structure was improved to improve the accuracy and speed of pedestrian detection. Two-stage algorithms mainly include R-CNN [30], Fast R-CNN [31], and Faster R-CNN [32]. Chen et al. [33] proposed a target detection method based on improved Faster R-CNN, and their method improved the accuracy of small-scale pedestrian detection with a mean accuracy improvement of 23.78% under the condition that the efficiency of small-scale pedestrian detection is equivalent. The literature [34] implements pedestrian detection for NIR nighttime images by improving the Faster R-CNN algorithm. In the literature [35], an improved YoLov3 model was proposed for detecting apples at different growth stages in orchards with fluctuating light and complex backgrounds, which can be applied to the actual environment of orchards. Compared with the two-stage algorithm, the one-stage algorithm is faster and has better real-time performance. YoLov4 is an excellent open-source target detection network with significant advantages in terms of speed and accuracy compared to contemporaneous target detection networks. However, it requires high hardware configuration and slower detection speed on small hardware platforms. And, YoLov4-tiny is widely used for detection on embedded platforms because of its advantages such as its small model and fast detection speed which exactly make up for the shortcomings of YoLov4. However, due to its simple network level and insufficient feature extraction ability, the detection effect is lower than YoLov4. Currently, the YoLo series framework has been updated to YoLov5, which has a network structure similar to YoLov4. Both use CSPDarknet53 as the backbone, PANET, and SPP as the neck, and the Head of YoLov3. In terms of performance, YoLov5 is slightly weaker than YoLov4 but is far more flexible and faster than YoLov4, offering a strong advantage in rapid deployment models.

The above-given pedestrian detection algorithms and frameworks have been hard to surpass for the overall detection of pedestrians, but problems such as occlusion by more than two people and false detection when the background is rich have not been solved in large scenes. To address the current challenges faced by target detection, this paper proposes a new MobileNet-YoLo pedestrian detection and recognition algorithm based on YoLov4-tiny, with the following main contributions.

1. To address the problem that the YoLov4-tiny network has simple layers and cannot extract more main features, the backbone feature extraction network is first replaced with MobileNetv3, and the output of the first two layers is subjected to MFF multiscale feature fusion to enrich semantic and spatial information, then the attention mechanism CBAM is added after the enhanced feature extraction network to obtain more effective local features and improve the detection accuracy, and finally the enhanced feature extraction network 3 × 3 ordinary convolution is replaced with deep separable convolution to further reduce the number of parameters.
2. In response to the slow convergence of the model and the existence of overfitting, ordinary data enhancement is proposed to expand the data online randomly to enrich the diversity of the data, and the k-means++ clustering algorithm is used to dynamically adjust the target detection anchor frame to avoid the problem of positive and negative sample imbalance.
3. To address the problem of scarcity of training samples and waste of resources, a migration learning approach is used to apply the trained model weights from the VOC2007 + 2012 dataset to the pedestrian dataset for retraining, which effectively solves the problem of sparse samples and greatly reduces the training time.

## 2. Principle of YoLov4-Tiny Algorithm

In target detection algorithms, the effectiveness of the detector directly affects the results of target detection and identification, while the speed of the detector and the size of the model is also critical to accomplishing real-time target detection. Most surveillance sites are embedded devices with low computational power, so it is not possible to deploy large-scale detection models. To reduce the computational cost and enhance the practicality, this paper selects YoLov4-tiny as the basic model for pedestrian detection.

YoLov4-tiny is designed based on the YoLov4 algorithm, which has faster target detection and the same prediction process as YoLov4. The algorithm first resizes the input images so that all input images have the same fixed size *A* × *A* (416 × 416) in this paper. The images of size *A* × *A* are divided into two scaled grids for prediction, *B*1 = *A*/26 and *B*2 = *A*/13. If the center coordinates of the target in the true size box fall in which grid, the target is predicted from this grid. The output of the network prediction is divided into two dimensions: one is the dimension of the extracted feature map, and the other is *n* × (5 + *N*), where *n* denotes the number of bounding boxes predicted by each grid, *n* in YoLov4-tiny is 3, *N* denotes the number of detected categories, and 5 denotes 4 coordinate information and 1 confidence level information. The model will output (*B*1 × *B*1 + *B*2 × *B*2) × *n* bounding boxes. Finally, the redundant bounding boxes are eliminated using the prediction confidence and nonmaximum suppression algorithms to obtain the final detected boxes of the model. The custom symbols of this study are shown in Table 1.

**Figure 1:**
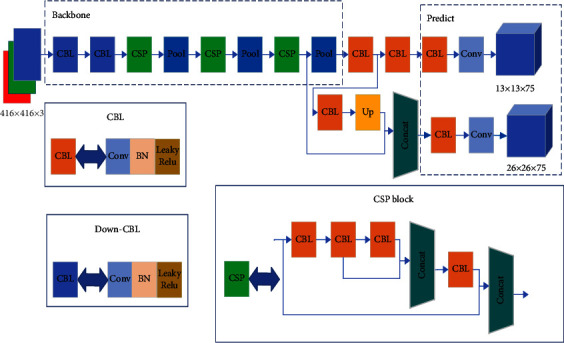
YoLov4-tiny network structure diagram.

YoLov4-tiny uses CSPDarknet53-tiny as the backbone feature extraction network to replace CSPDarknet53 used in YoLov4. In the enhanced feature extraction module, features at different scales are extracted and fused using the feature pyramid network FPN to improve the accuracy of target detection. Finally, two different scales of 13 × 13 and 26 × 26 feature maps are used to predict the detection results. The structure of the YoLov4-tiny network is shown in Figure 1.

## 3. A MobileNet-YoLo Pedestrian Detection Algorithm

**Figure 2:**
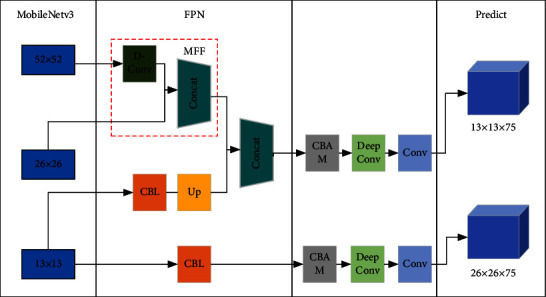
Network framework of MobileNet-YoLo model.

Based on the YoLov4-tiny target detection algorithm, this study first replaces the backbone feature extraction network with MobileNetv3 and fuses the first two of the three output layers of MobileNetv3 through the MFF module, then adds the attention mechanism CBAM after the enhanced feature extraction network to obtain more effective local features and improve the detection accuracy, and finally replaces the 3 × 3 ordinary convolutions after the enhanced feature extraction network with a deep separable convolution to further reduce the number of parameters and better achieve the embedded possibility. The MobileNet-YoLo network framework is shown in Figure 2.

### 3.1. YoLov4-Tiny Fusion MobileNetv3

MobileNetv3 integrates the deeply separable convolution of MobileNetv1 [36] and the linear bottleneck inverse residual structure of MobileNetv2 [37] with a lightweight attention module. To better compare the role provided by the MobileNet model for the YoLov4-tiny backbone network, the number of parametric Params is selected as an evaluation metric. Among them, the number of parametric Params directly determines the size of the deep learning model, which can be equated to the space complexity of the algorithm. As can be seen from Table 2, although MobileNetv2 has the smallest number of parameters, the accuracy is more important under the premise of ensuring a low number of parameters, so MobileNetv3, which has a slightly higher number of parameters, is selected as the backbone feature extraction network of YoLov4-tiny.

MobileNetv3 uses lightweight modules with a small number of parameters and can effectively extract feature information by extracting feature images layer by layer, so its advantages are utilized to replace CSPDarknet53-tiny and improve detection accuracy by utilizing its optimized model's feature extraction capability while ensuring fast detection. In this paper, the output of MobileNetv3 layer 7, layer 13, and layer 16 are extracted the output of layer 7, layer 13, and layer 16 of MobileNetv3 is extracted, and layer 7 and layer 13 are multiscale fused by MFF, and then the fusion result is used as the input of the enhanced feature extraction network together with layer 16, and the corresponding output sizes and the number of channels are 52 × 52 × 40, 26 × 26 × 112 and 13 × 13 × 160, respectively. The overall structure of MobileNetv3 corresponding to this paper is shown in Table 3.

### 3.2. YoLov4-Tiny Fusion Attention Mechanism

**Figure 3:**
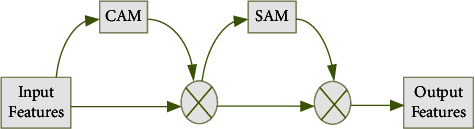
CBAM module structure.

The convolutional block attention module (CBAM) [38] is a lightweight general-purpose module for feedforward convolutional neural networks that can be seamlessly integrated into any CNN architecture with a negligible number of parameters and can be trained end-to-end with the basic CNN. CBAM has two submodules. Channel attention module (CAM) and spatial attention module (SAM). Among them, CAM focuses on the channel features that play a decisive role in the final prediction, and SAM focuses on the location information that plays a decisive role in the final prediction. The model is shown in Figure 3. The input features are passed through the channel attention CAM and multiplied by the original features, and the new feature map obtained is multiplied by the spatial attention SAM to finally obtain the adjusted feature map.

The most important function of the attention mechanism is to reconstruct the attention points of the feature map, highlight the important information in the feature map, and suppress the general information. Therefore, in this paper, we combine the backbone feature extraction network and the enhanced feature extraction network and then fuse CBAM for secondary enhancement of the features to strengthen the information.

### 3.3. YoLov4-Tiny Fusion Depth Separable Convolution

**Figure 4:**
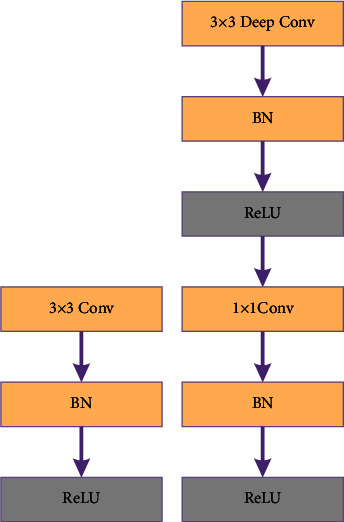
Standard convolution (a) and depth-separable convolution (b).

In the processing of images containing a lot of noise and information, depth-separable convolution has the advantages of fewer parameters and shorter training time compared with the traditional convolution operation. It can extract feature information in an image more quickly with the same accuracy. In this network, the convolution operation uses 1 × 1 convolution and 3 × 3 convolutions. Standard convolution combines filtering and input into a new set of outputs, while depth-separable convolution is a combination of depthwise convolution (DW) and pointwise convolution (PW), one for filtering and the other for merging to extract features. DW is the convolution kernel corresponding to the input channels. Each input channel is convolved independently in space; PW is used to combine the output of DW; i.e., each channel is convolved with the same number of channels separately, and then the result of the first step is combined with the 1 × 1 convolution across channels. The convolution operation is shown in Figure 4.

In the depth-separable convolution of this paper, the different input channels are first convolved using DW, and then the outputs are combined using PW. The overall effect is the same as that of standard convolution, but it will greatly reduce the computation and model parameters and is more suitable for extracting pedestrian features.

### 3.4. YoLov4-Tiny Fusion Multiscale Features

**Figure 5:**
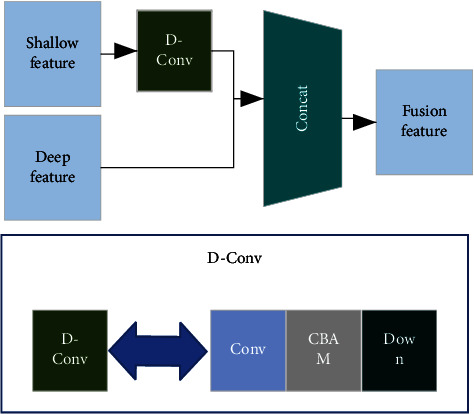
Structure of MFF.

Inspired by FPN, the multiscale feature fusion (MFF) model is proposed by applying its idea of multiscale feature fusion. MFF is a module that fuses different levels of feature maps, and its structure is shown in Figure 5. Since the resolution of the image decreases gradually when the network performs feature learning, generally the high-resolution shallow features are close to the input while the low-resolution deep features are close to the output. Therefore, the feature map of 52 × 52 size and channel number 40 generated by the output of layer 7 of the backbone network is first 1 × 1 convolutionally up-dimensioned to make its channel number 112, the purpose of up-dimensioning is to obtain more feature information, and then the obtained feature map is enhanced feature extraction by the attention mechanism module CBAM to strengthen the feature expression capability, and finally, it is down-sampled by 0.5 times to make branch sizes match each other to obtain a branch in the FPN; then, because the deep network has more effective information, the feature map of 26 × 26 size and 112 channels generated from the output of layer 13 of the backbone network is directly spliced and fused with the shallow feature branch. The use of MFF can obtain richer semantic and spatial information and effectively improve the prediction accuracy of the network model.

### 3.5. MobileNet-YoLo Loss Function

To obtain accurate location and category information in complex environments, the loss function [39] needs to be optimized to balance the training error of the prediction frame, confidence, and category.

In this study, complete intersection over union (CIoU) is used as the loss function. Compared with the traditional loss function, CIoU is used to measure the distance and overlap between the target and prediction frames, coordinate the distance, overlap ratio, ratio, and penalty term between the target and anchor frames, make the regression of the target frame more stable, and do not have divergence and other problems during the training process like the intersection and merge ratio (IoU) [40] and the full intersection and merge ratio (GIoU) [41] and use the aspect ratio of the prediction frame as a penalty term, the effect is more stable.

The loss function should consider three geometric factors, i.e., overlap area, distance, and aspect ratio. The total loss value is shown in the following equation:

(1) $$\begin{array}{c} L=S\left(E,E^{gt}\right)+D\left(E,E^{gt}\right)+V\left(E,E^{gt}\right)\text{,} \end{array}$$

where *S*, *D*, and *V* denotes the overlap area, distance, and aspect ratio, *E*, *E* ^*gt*^, respectively, for the 2 prediction frames. And, the IoU and GIoU losses are considered only for the overlap area, as shown in the following equation:

(2) $$\begin{array}{c} S=1-IoU\text{.} \end{array}$$

The normalized centroid distance is used to measure the distance of the 2 prediction frames, as shown in the following equation:

(3) $$\begin{array}{c} D=\frac{\rho^{2}\left(p,p^{gt}\right)}{c^{2}}\text{,} \end{array}$$

where *p*=[*x*, *y*]^*T*^ and *p* ^*gt*^=[*x* ^*gt*^, *y* ^*gt*^]^*T*^ are the centroids of boxes *E* ^*gt*^, *c* is the diagonal length of the box, and *ρ* is designated as the Euclidean distance.

The consistency of the aspect ratio is achieved as shown in the following equation:

(4) $$\begin{array}{c} V=\frac{4}{\pi^{2}}{\left(\arctan\frac{w^{gt}}{h^{gt}}-\arctan\frac{w}{h}\right)}^{2}\text{,} \end{array}$$

where *w* and *w* ^*gt*^ are the widths of boxes *E* and *E* ^*gt*^, and *h* and *h* ^*gt*^ are the heights of boxes *E* and *E* ^*gt*^. Finally, the loss function CIoU of the complete IoU is obtained, as shown in the following equation:

(5) $$\begin{array}{c} L_{CIoU}=1-IoU+\frac{\rho^{2}\left(p,p^{gt}\right)}{c^{2}}+\alpha V\text{,} \end{array}$$

where *α* is a trade-off parameter as shown in the following equation:

(6) $$\begin{array}{c} \alpha =\left\{\begin{array}{c} \frac{V}{\left(1-IoU\right)+V},IoU\geq 0.5\text{,}\\ 0,IoU<0.5\text{.} \end{array}\right. \end{array}$$

### 3.6. Resetting the Target Detection Anchor Frame

In the anchor frame-based target detection network, the reasonableness of the anchor frame setting is crucial to the performance of the final model. If the size of the anchor frame does not match the size of the object under test, the number of positive samples of the anchor frame may be very small, leading to a large number of missed and false detections. Most target detection networks use default generic anchor frame parameters to fit a generic public dataset. To avoid the problem of positive and negative sample imbalance caused by using generic anchor frames on a specific dataset, this paper uses the k-means++ clustering algorithm [42] to regenerate new anchor frame parameters based on the clustering centers and data frame distributions. The clustering distance reflects the error between the candidate and real boxes by IoU. The distance equation is shown in the following equation:

(7) $$\begin{array}{c} D\left(box,center\right)=1-IoU\left(box,center\right)\text{,} \end{array}$$

where the center is the center of all clusters, the box is the sample clustering result, and IoU denotes the intersection and merging ratio of all centers to all boxes.

## 4. Experimental Results and Analysis

The experimental hardware configuration is AMD Ryzen 5 1600 CPU, 8 GB RAM, NVIDIA GeForce GTX 1650 DDR6 graphics card with 4 Gb video memory, Ubuntu 18.04, 64-bit software environment, PyTorch 1.2.0 deep learning framework, CUDA parallel computing framework 11.

### 4.1. Target Detection Dataset

The VOC dataset is one of the most commonly used standard datasets in the field of target detection. Almost all papers in the detection direction give their results of training and evaluation on the VOC dataset. Therefore, the VOC2007 + 2012 dataset is selected for this study to validate the reliability of the model. This dataset has 20 categories with a total of 21,503 images, 17,416 in the training set, 1,936 in the validation set, and 2,151 in the test set, where the ratio of the training set to the validation set is 9 : 1.

The COCO dataset is a large and rich dataset of object detection, segmentation, and captioning. This dataset mainly intercepts objects in images from complex everyday scenes and calibrates the position by precise coordinates. Therefore, images with a high number of pedestrians present in the COCO dataset are selected for the pedestrian detection and recognition task in this study. The initial screening using the tags of the dataset, the initial selection of images with more negative samples, mislabeled or difficult to recognize by the naked eye alone, is not conducive to the realization of this pedestrian detection task, so the more than 80,000 images screened are then manually selected to match the images and tags of this experiment. The selected dataset contains only one category of pedestrians, with a total of 21,100 images, 17,091 in the training set, 1,899 in the validation set, and 2,110 in the test set, which is also 9 : 1 for the training set and the validation set. The rest of the untested dataset does not contain many pedestrian data, so it is not considered in this study.

After clustering, [(16, 29), (31, 88), (54, 47), (79, 139), (163, 227), (338, 330)] are used for the training of VOC2007 + 2012 dataset, and [(6, 13), (15, 33), (32, 65), (57, 147), (110, 256), (233 342)] for the training of the COCO pedestrian dataset.

### 4.2. Target Detection Evaluation Metrics

In this paper, the mean average accuracy (mAP) and the number of frames transmitted per second (Speed), and the total number of parameters (Params) are used as evaluation metrics for the detection accuracy and detection speed of the algorithm and the model size, respectively. In the target detection task, mAP is the mean value of the average accuracy (AP) for each type of target, as shown in the following equation:

(8) $$\begin{array}{c} mAP=\frac{1}{N}\overset{N}{\underset{i=1}{\sum }}AP_{i}\text{,} \end{array}$$

where *N* is the number of categories and *i* denotes a particular category. The *AP* for a particular category *i* is calculated as follows:

(9) $$\begin{array}{c} AP_{i}=\overset{1}{\underset{0}{\int }}P\left(R\right)dR\text{,} \end{array}$$

where *P*(*R*) is the mapping relationship between precision (*P*) and recall (*R*), which is often represented by a *P-R* curve, and the area of the region below the curve is the *AP* value for that category. Precision and recall are calculated as follows:

(10) $$\begin{array}{c} P_{i}=\frac{TP}{\left(TP+FP\right)}\text{,}\\ R_{i}=\frac{TP}{\left(TP+FN\right)}\text{,} \end{array}$$

where TP denotes the number of samples where both the detection category and true label are *i*, FP denotes the number of samples where detection category is *i* and true label is not *i*, and FN denotes the number of samples where detection category is not *i* but the true label is *i*.

### 4.3. Model Training Strategy and Loss Function

The use of an appropriate model training strategy plays a crucial role in the effectiveness of the experiment, and Table 4 compares the effects of the two data enhancement methods and other training strategies on the mAP values of this model.

This experiment was validated on the VOC2007 + 2012 dataset without loading any model weights, with a network input size of 416 × 416, optimizer selection Adam, 800 training rounds, batch size 8, and an initial learning rate of 0.0001.

The idea of the Mosaic data enhancement method is to randomly crop four images and stitch them onto a single image as training data, which has the advantage of increasing the diversity of data and enriching the background of the images. However, Mosaic data augmentation may not be effective for all data, especially for some datasets with larger targets, it does not make much sense, and it is far from the real distribution of natural images when generating training images. However, since data enhancement can well moderate the errors of feature extraction due to angle, brightness, and other factors, this paper proposes an Ordinary common data enhancement method, including scaling, distorting, cropping, flipping, HSV color gamut transformation of the image, and then adjusting the a priori frame of the modified image to match the enhanced data. The experimental results show that online random adjustment of the dataset by the Ordinary data enhancement approach effectively expands the dataset and improves the detection accuracy while increasing diversity.

Cosine_lr cosine annealing learning rate can be dynamically adjusted by the cosine function, and as the Loss value gets closer to flat, the learning rate will become smaller to make the model as close to the global optimal solution as possible, among which the best effect is achieved for the experiment when using Cosine_lr and normal data augmentation, and the mAP value of the model reaches 73.17%. While Label_smoothing label smoothing can make a corresponding penalty to classification accuracy and prevent the model from overfitting, the actual effect is not obvious.

Table 5 shows the accuracy comparison of the MobileNet-YoLo regression loss function using different bounding boxes. Compared with IoU, DIoU loss can better establish the connection between the four boundary points and fully reflect the overlap of the bounding boxes. CIoU considers the diagonal length of the center point of the bounding box, the length and width of the bounding box, and the diagonal length of the minimum closed rectangle, and obtains the highest accuracy improvement.

### 4.4. MobileNet-YoLo Ablation Experiment

This experiment is based on Section 4.3, where the general data enhancement and Cosine_lr cosine annealing learning rate are applied to the ablation experiment with the same data set and parameters.

Table 6 shows the MFF ablation experiments. To match the high and low branch sizes, both 0.5 times downsampling was performed in the shallow feature branch to compare the effects of 1 × 1 convolution and the attention mechanism CBAM on the performance of the MFF model. When only downsampling is performed, the model performance is slightly improved by fusing the high and low branches to obtain features at different levels. Then, after obtaining more channel information by 1 × 1 convolutional up-dimensioning, the mAP value is improved by 0.17%, the detection speed is slightly decreased by 0.45 FPS, and the Params value is improved by 0.05 MB. And, after adding the attention mechanism CBAM, it can play the role of enhancing the feature expression and further improving the model accuracy. Combining these two improvements, the mAP value is 1.68% higher compared to the original model, while the detection speed only decreases by 2.62 FPS, and the overall Params of the model is 5.02 MB. Thus, it can be seen that the MFF model effectively improves the model accuracy while ensuring that the detection speed and the number of parameters remain unchanged.

Table 7 compares the effects of four improvement strategies, MobileNetv3, MFF, attention mechanism, and deep separable convolution, on the MobileNet-YoLo model. For the mAP value, the mAP value of the original model is 68.17%. Although the mAP value decreases by 0.65% after adopting deep separable convolution, its detection speed increases by 7.18 FPS, and Params decreases by 1.83 MB, which provides the possibility of a better-grounded application of the model. Then, after using the MobileNetv3 backbone feature extraction network, the mAP value improved by 2.81% and the Params of the model further decreased by 0.94 MB, and although the detection speed showed a substantial decrease, it still reached the real-time level. In MobileNetv3, after using the MFF structure to fuse the first two output layers, the mAP value increased by 0.71%, the Params of the model increased slightly by 0.04 MB, and the detection speed decreased slightly by 1.83 FPS. Adding the attention mechanism CBAM after strengthening the feature extraction network can strengthen the valid information and suppress the redundant information, which can improve the detection and recognition accuracy. Improve the accuracy of detection and recognition, and assure the detection effectiveness of the MobileNet-YoLo model. Combining these four improvements, the mAP improves 5.00% over the original model, the Speed stays at the level of real-time detection, reaching 73.39 FPS, and the overall Params is 3.23 MB, which is 2.69 MB lower than the original model.

### 4.5. MobileNet-YoLo Comparison Experiments

To verify the detection effectiveness of the MobileNet-YoLo model, YoLov3, YoLov4, MobileNetv3-YoLov3, MobileNetv3-YoLov4, YoLov4-tiny, YoLov5s, and YoLov5x models were used for comparison, and with the same training parameters as well as training techniques, the models have all converged. And, the mAP values were calculated for all categories detected in the VOC2007 + 2012 dataset, and the results are shown in Table 8.

For MobileNetv3-YoLov3 and MobileNetv3-YoLov4, which also use the MobileNetv3 backbone feature extraction network, MobileNet-YoLo outperforms these two models in terms of mAP value, Speed, and Params. And, comparing with YoLov3, MobileNet-YoLo is much faster than YoLov3 with 14.25 FPS in mAP, although it is 5.13% lower in mAP, and the number of parameters is about 1/19 times of YoLov3. The same is true for YoLov4, which achieves a maximum mAP of 82.30%, but is not suitable for online pedestrian detection and recognition tasks due to a large number of parameters. As the latest model of the YoLo series, MobileNet-YoLo outperforms YoLov5s in terms of accuracy, speed, and several model parameters when compared with YoLov5s. By comparing YoLov3, YoLov4, and YoLov5, it can be concluded that YoLov4 is the best in terms of overall performance, but YoLov5 is comparable to MobileNet-YoLo in terms of flexibility. This is sufficient to show that MobileNet-YoLo has met the requirements of online target detection and recognition tasks in terms of detection accuracy, speed, and the number of parameters.

Unlike previous work, which designed the model structure and trained the model using positive and negative samples; here, the classifier model is not completely retrained in this paper. Since retraining the model requires labeling a large number of samples and will result in the waste of previous knowledge, to solve the problems of insufficient samples and data reusability, the pedestrian classification weights obtained on the VOC2007 + 2012 task are used for the existing pedestrian detection and recognition task using transfer learning techniques to increase the model training effect, and the results are shown in Table 9.

**Figure 6:**
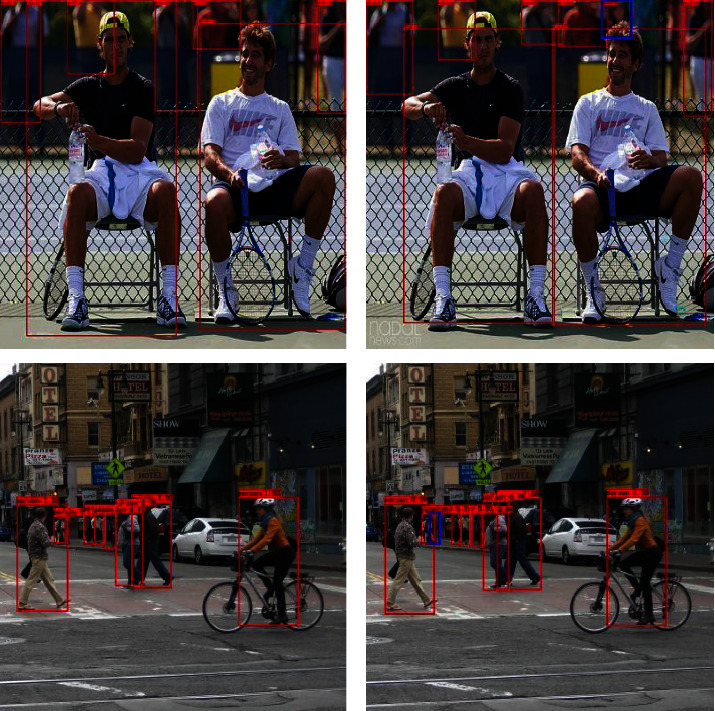
Detection effects of YoLov4-tiny (a) and MobileNet-YoLo (b).

To visually demonstrate the differences between the original YoLov4-tiny algorithm and the proposed MobileNet-YoLo detection algorithm, two detection images are selected for comparative analysis. The detection results of YoLov4-tiny and MobileNet-YoLo are shown in Figure 6, where the blue area shows the differences in the recognition effects of the two detection algorithms.

As can be seen from Figure 6, for the first image, the pedestrians located in the distant and blurred position of the image can also be accurately detected and recognized by MobileNet-YoLo. In the second image, MobileNet-YoLo can also accurately locate the two occluded pedestrians. Therefore, compared with the YoLov4-tiny detection algorithm, the MobileNet-YoLo algorithm is still able to recognize relatively distant targets, but the original YoLov4-tiny is unable to detect them, and the recognition accuracy of MobileNet-YoLo is 3.86% higher than that of YoLov4-tiny improved by 3.86%.

## 5. Conclusions

In this paper, the MobileNet-YoLo model is proposed to address the problems of simple YoLov4-tiny network hierarchy, slow convergence speed, and lack of training samples. The experimental results show that MobileNet-YoLo has met the requirements of online target detection and recognition tasks in terms of detection accuracy, speed, and the number of parameters. However, there is still a 9.13% difference in accuracy with the comparison model YoLov4. The next work will continue to improve the model to achieve the possibility of high accuracy and fast detection. And, the MobileNet-YoLo detection algorithm is still able to accurately identify targets that are relatively far away, providing the possibility of online detection.

## Figures and Tables

**Table 1:** Symbol table.

*A*	Input image size
*B*1 and *B*2	Prediction box size
*n*	Number of bounding boxes per grid prediction
*N*	Number of detected categories
*L*	Total loss
*S*	Overlap area
*D*	Distance
*V*	Aspect ratio
*E* and *E* ^*gt*^	Prediction box
*p* and *p* ^*gt*^	The center point of the box *E* and box *E* ^*gt*^
*c*	The diagonal length of the frame
*ρ*	Euclidean distance
*w* and *w* ^*gt*^	The width of box *E* and box *E* ^*gt*^
*h* and *h* ^*gt*^	The height of box *E* and box *E* ^*gt*^
*α*	Weighing parameters
Center	Center of all clusters
Box	Sample clustering results
IoU	The intersection ratio of all centers to all boxes
mAP	Average precision means
Speed	Transfer frames per second
Params	A total number of participants
AP	Average accuracy
*i*	A category
*P*	Accuracy
*R*	Recall rate
*P*(*R*)	Mapping between precision and recall
TP	Several samples for which both the detection category and the true label are *i*
FP	Several samples with detection category *i* and true label not *i*
FN	Detect the number of samples with category not *i* but with true label *i*
*L*_IoU_, *L*_DIoU_, and *L*_CIoU_	Loss function

**Table 2:** Comparison of model parameters.

Models	Params (MB)
YoLov4-tiny	5.92
YoLov4-tiny-MobileNetv1	6.22
YoLov4-tiny-MobileNetv2	**3.68**
YoLov4-tiny-MobileNetv3	4.68

**Table 3:** MobileNetv3 structural parameters.

Input^1^	Operator^2^	Exp size^3^	Out^4^	SE^5^	NL^6^	S^7^
416^2^ × 3	Conv2d	—	16	—	HS	2
208^2^ × 16	Bottleneck, 3 × 3	16	16	—	RE	1
208^2^ × 16	Bottleneck, 3 × 3	64	24	—	RE	2
104^2^ × 24	Bottleneck, 3 × 3	72	24	—	RE	1
104^2^ × 24	Bottleneck, 5 × 5	72	40	✔	RE	2
52^2^ × 40	Bottleneck, 5 × 5	120	40	✔	RE	1
52^2^ × 40	Bottleneck, 5 × 5	120	40	✔	RE	1
52^2^ × 40	Bottleneck, 3 × 3	240	80	—	HS	2
26^2^ × 80	Bottleneck, 3 × 3	200	80	—	HS	1
26^2^ × 80	Bottleneck, 3 × 3	184	80	—	HS	1
26^2^ × 80	Bottleneck, 3 × 3	184	80	—	HS	1
26^2^ × 80	Bottleneck, 3 × 3	480	112	✔	HS	1
26^2^ × 112	Bottleneck, 3 × 3	672	112	✔	HS	1
26^2^ × 112	Bottleneck, 5 × 5	672	160	✔	HS	2
26^2^ × 160	Bottleneck, 5 × 5	960	160	✔	HS	1
13^2^ × 160	Bottleneck, 5 × 5	960	160	—	HS	1

^1^Input represents the shape change of each feature layer; ^2^Operator represents the block structure that each feature layer is about to experience; ^3^Exp size, ^4^Out represent the number of channels that rise in the inverse residual structure within the neck, and the number of channels in the feature layer at the time of input to the neck, respectively; ^5^SE represents whether the attention mechanism is introduced at this layer; ^6^NL represents the type of activation function, HS represents h-swish, and RE represents RELU; ^7^S represents the step length used for each block structure.

**Table 4:** mAP values of different training skills in MobileNet-YoLo.

Mosaic	Ordinary	Cosine_lr	Label_smoothing = 0.01	mAP (%)
✔		✔		71.79
✔			✔	48.43
✔		✔	✔	71.10
	✔	✔		**73.17**
	✔		✔	49.58
	✔	✔	✔	72.32

**Table 5:** MobileNet-YoLo loss function.

Loss function	mAP (%)
*L*_IoU_	70.26
*L*_DIoU_	71.54
*L*_CIoU_	**73.17**

**Table 6:** MFF ablation experiments.

Conv	CBAM	mAP (%)	Speed (FPS)	Params (MB)
		70.01	**70.98**	**4.86**
✔		70.18	70.53	4.91
	✔	70.54	69.89	4.97
✔	✔	**71.69**	68.36	5.02

**Table 7:** MobileNet-YoLo ablation experiment.

MobileNetv3	MFF	CBAM	DS Conv	mAP (%)	Speed (FPS)	Params (MB)
				68.17	107.76	5.92
			✔	67.52	**114.94**	4.09
✔			✔	70.33	77.37	**3.15**
✔	✔		✔	71.04	75.54	3.19
		✔	✔	71.22	109.21	4.14
✔	✔	✔	✔	**73.17**	73.39	3.23

**Table 8:** Comparison of target detection results of different algorithms.

Models	Basic network	mAP (%)	Speed (FPS)	Params (MB)
YoLov3	Darknet53	78.30	14.25	61.63
YoLov4	CSPDarknet53	**82.30**	20.37	64.04
MobileNetv3-YoLov3^1^	MobileNetv3	69.94	46.92	23.29
MobileNetv3-YoLov4^2^	MobileNetv3	71.87	49.04	11.41
YoLov4-tiny	CSPDarknet53_tiny	68.17	**107.76**	5.92
YoLov5s	CSPDarknet53	72.43	67.14	7.12
YoLov5x	CSPDarknet53	81.18	10.09	87.37
MobileNet-YoLo	MobileNetv3	73.17	73.39	**3.23**

^1^MobileNetv3-YoLov3 and^2^MobileNetv3-YoLov4 are models for adjusting the backbone feature extraction network.

**Table 9:** Experimental comparison between different models.

Models	mAP (%)
MobileNetv3-YoLov3	75.04
MobileNetv3-YoLov4	73.56
YoLov4-tiny	72.81
YoLov5s	75.43
MobileNet-YoLo	**76.67**

## Data Availability

The data used to support the study are included in the paper.
